# Contractile Protein Expression and Phosphorylation and Contractility of Gastric Smooth Muscles from Obese Patients and Patients with Obesity and Diabetes

**DOI:** 10.1155/2018/8743874

**Published:** 2018-05-31

**Authors:** Wen Li, Kent C. Sasse, Yulia Bayguinov, Sean M. Ward, Brian A. Perrino

**Affiliations:** ^1^Department of Physiology and Cell Biology, University of Nevada, Reno School of Medicine, Reno, NV 89557, USA; ^2^Sasse Surgical Associates, Reno NV 89502, USA; ^3^Renown Regional Medical Center, Reno, NV 89502, USA

## Abstract

Ingested food is received, mixed, and ground into chyme by distinct gastric motility patterns. Diabetes impairs gastric muscle function, but the mechanisms underlying diabetes-induced gastric muscle dysfunction are unknown. Here, we compared the expression and phosphorylation of Ca^2+^ sensitization and contractile proteins in human gastric muscles from obese nondiabetic and diabetic patients. We also compared the spontaneous phasic contractions and the contractile responses evoked by electrical field stimulation of cholinergic motor neurons. Fundus and antrum muscles were obtained from sleeve gastrectomies and were used in in vitro myobath contractile studies and for capillary electrophoresis and immunodetection of *γ*-actin, CPI-17, pT38-CPI-17, MYPT1, pT853-MYPT1, pT696-MYPT1, myosin light chain (MYL9), pS19-MYL9, myosin light chain kinase (MYLK), protein phosphatase-1*δ* (PP1*δ*), and Rho-associated kinase (ROCK2). In diabetic fundus muscles, MYLK, ROCK2, and PP1*δ* expression was unchanged; MYPT1 and CPI-17 expression was decreased; and the pT853/MYPT1 and pT38/CPI-17 ratios, but not the pT696/MYPT1 ratio, were increased. Although MYL9 expression was increased, the pS19/MYL9 ratio was unchanged in diabetic fundus muscles. In diabetic antrum muscles, MYLK and MYL9 expression was unchanged, but ROCK2, CPI-17, and PP1*δ* expression was decreased. The pT38/CPI-17 ratio was unchanged, while the pS19/MYL9, pT853/MYPT1, and pT696/MYPT1 ratios were decreased, consistent with the reduced ROCK2 expression. The frequencies of spontaneous phasic contractions from nondiabetic and diabetic gastric fundus and antrum muscles did not significantly differ from each other, regardless of age, sex, or diabetic status. The fold increases in the contractions of diabetic fundus and antrum muscles in response to increased frequencies of electrical field stimulation were significantly lower compared to nondiabetic fundus and antrum muscles. The altered contractile responses and the protein expression and phosphorylation in gastric muscles of obese patients with diabetes illustrate the importance of understanding how smooth muscle Ca^2+^ sensitization mechanisms contribute to gastric motility.

## 1. Introduction

The stomach carries out the second phase of digestion, which involves accommodation, chemical and mechanical disruption of solids into chyme, and controlled emptying into the duodenum. To carry out these distinct functions, the stomach is composed of anatomical regions with different motility patterns [1]. The fundus is responsible for gastric accommodation and the sustained contractions that move ingested food into the corpus and antrum where strong phasic contractions underlie the peristaltic digestive activity of the stomach [2]. The pylorus regulates delivery of chyme to the duodenum [1].

The primary trigger of gastrointestinal (GI) smooth muscle contraction is an increase in intracellular Ca^2+^ [Ca^2+^]_I_, which activates the calmodulin-dependent myosin light chain kinase (MYLK) to phosphorylate myosin regulatory light chain (MYL9) at S19, stimulating myosin ATPase activity to activate cross-bridge cycling and contraction [3, 4]. Termination of the contractile stimulus leads to a decrease in [Ca^2+^]_i_, thereby decreasing MYLK activity. Consequently, MYL9 is dephosphorylated by myosin light chain phosphatase (MLCP), leading to relaxation [5]. Inhibiting MLCP while activating MYLK generates greater force by further increasing MYL9 phosphorylation [6]. This phenomenon is termed “Ca^2+^ sensitization of the contractile apparatus,” to describe the increased Ca^2+^ sensitivity of the contractile response [7]. MLCP activity is regulated by upstream signaling pathways [8–10]. Phosphorylation of the protein kinase C- (PKC-) potentiated phosphatase inhibitor protein-17 kDa (CPI-17) greatly increases its inhibition of MLCP [8–10]. Phosphorylation of the myosin phosphatase targeting subunit of MLCP (MYPT1) at T696 (human isoform numbering) by Rho-associated kinase (ROCK2) inhibits MLCP activity [11, 12]. ROCK2 also phosphorylates MYPT1 T853 and inhibits MLCP, but this phosphorylation does not appear to inhibit MLCP in vivo [13, 14]. However, ROCK2 activity is clearly required for Ca^2+^ sensitization and augmented contraction [13]. Thus, the level of MYPT1 T853 phosphorylation indicates ROCK2 activity and Ca^2+^ sensitization in smooth muscles [13].

Previously, we found that the protein expression levels of ROCK2, MYPT1, and CPI-17 relative to MYLK and MYL9 are significantly higher in murine gastric fundus muscles compared to antrum muscles [15]. Gastric fundus muscles have high basal levels of CPI-17, MYPT1, and MYL9 phosphorylation, and CPI-17 phosphorylation is increased by cholinergic motor neurotransmission [15, 16]. Gastric dysmotility is observed in animal models of diabetes including streptozotocin-induced diabetes in rats and in genetically induced diabetes of *NOD*, *db/db*, and *ob/ob* mice [17–19]. We found that ROCK2 expression and basal and agonist-induced phosphorylation of MYPT1 and MYL9, but not CPI-17, was decreased in gastric antrum muscles of *ob/ob* mice, a model of obesity and type 2 diabetes [20].

Due to the functional diversity of gastric smooth muscles, the relative importance of CPI-17 and MYPT1 phosphorylation to myofilament Ca^2+^ sensitization and smooth muscle contractile patterns is likely different between the functional regions of the stomach. Each region of the stomach likely has different requirements for how MLCP activity and the Ca^2+^ sensitivity of contraction are regulated during healthy gastric functioning. In addition, the function of Ca^2+^ sensitization mechanisms may be adversely altered in pathophysiological conditions that affect gastric motility [21]. However, most studies of gastric muscles have used animal models, with relatively few studies examining the contractile properties and expression of Ca^2+^ sensitization proteins of human gastric smooth muscles [22–25]. Significant ultrastructural changes to gastric smooth muscle cells are observed in gastric biopsy specimens from patients with diabetic gastroparesis, suggesting that impaired gastric smooth muscle function from long-standing diabetes could contribute to the pathophysiology of diabetic gastroparesis [26–28]. Thus, in the present study, we sought to compare the contractile properties and determine the expression and phosphorylation levels of Ca^2+^ sensitization and contractile proteins in human gastric fundus and antrum smooth muscles, using resected stomach specimens obtained from nondiabetic and diabetic patients undergoing vertical sleeve gastrectomies.

## 2. Materials and Methods

### 2.1. Human Stomach Smooth Muscles

**Figure 1:**
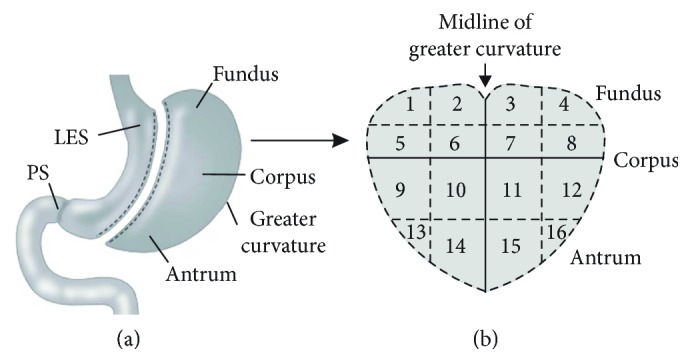
Diagram and map of anatomical regions of the resected stomach from vertical sleeve gastrectomy. (a) Sketch of the anatomical regions of the resected stomach from a typical vertical sleeve gastrectomy. (b) Schematic map used to demarcate the corresponding anatomical regions of the resected stomach when laid out flat.

The use of human resected stomach tissues was approved by the Human Subjects Research Committees at the Renown Regional Medical Center and the Biomedical Institutional Review Board at the University of Nevada, Reno, and was conducted in accordance with the Declaration of Helsinki (revised version, October 2008, Seoul, South Korea). All patients provided written informed consent. Resected stomach specimens were acquired immediately after surgery from patients undergoing vertical sleeve gastrectomy.  Table 1 shows the clinical characteristics of the 14 nondiabetic and 14 diabetic patients from which the resections were obtained. The resected stomach tissue was placed into ice-cold Krebs–Ringer buffer (KRB; composition (in mM): NaCl 118.5, KCl 4.5, MgCl_2_ 1.2, NaHCO_3_ 23.8, KH_2_PO_4_ 1.2, dextrose 11.0, and CaCl_2_ 2.4; when bubbled with 97% O_2_–3% CO_2_ at 37°C, the pH of KRB was 7.3–7.4.) for transport to the laboratory. The gastric fundus region was identified by its bulbous appearance, and the gastric antrum region was identified by its narrow tapered shape. The resected stomach tissues were opened along the staples, laid out flat, and pinned to a Sylgard-lined dish containing oxygenated KRB. The mucosa and submucosa were removed by sharp dissection. Gastric fundus and antrum muscles were mapped as indicated in Figure 1 and obtained from regions 1–4 and regions 13–16, respectively [24]. Rectangular strips (∼2 mm × 10 mm × 2 mm) of full thickness muscle were used for the protein expression and phosphorylation studies. Muscle strips were pretreated with 1 *μ*M carbachol for 5 min at 37°C in oxygenated KRB, and three 1 min washes with KRB, to remove any residual curariform neuromuscular paralytics [29]. The muscle strips were equilibrated for 1 hour in 37°C oxygenated KRB, followed by an additional 1-hour incubation in 37°C oxygenated KRB with 0.3 *μ*M tetrodotoxin. For automated capillary electrophoresis and Western blotting, the muscles were submerged into ice-cold acetone/10 mM DTT/10% (*w*/*v*) trichloroacetic acid for 2 min, snap-frozen in liquid N_2_, and stored at −80°C for subsequent Wes analysis [15, 30].

### 2.2. Automated Capillary Electrophoresis and Chemiluminescent Western Blotting

Muscles were thawed on ice, followed by three 1 min washes in ice-cold acetone/DTT and a 2 min wash in ice-cold lysis buffer (mM; 50 Tris–HCl pH 8.0, 60 *β*-glycerophosphate, 100 NaF, 2 EGTA, 25 sodium pyrophosphate, and 1 DTT, with 0.5% NP-40, 0.2% SDS, and a protease inhibitor tablet (Roche, Indianapolis, IA, USA)) [15, 30]. Each tissue was homogenized in 0.5 mL lysis buffer using a bullet blender and centrifuged at 16000 ×g at 4°C for 10 min, and the supernatants were aliquotted and stored at −80°C. Protein concentrations were determined by Bradford assay, using bovine *γ*-globulin as the standard [15]. Analysis of protein expression and phosphorylation was performed according to the Wes User Guide using a Wes instrument from ProteinSimple (San Jose, CA, USA). Protein samples were mixed with fluorescent 5x Master Mix and incubated at 95°C for 5 min. The samples were loaded into the Wes plate (Wes 12–230 kDa prefilled plates with split buffer) along with a biotinylated protein ladder, blocking reagent, primary antibodies, ProteinSimple HRP-conjugated anti-rabbit secondary antibody, luminol peroxide, and washing buffer. The plates and capillary cartridges were loaded into the Wes for electrophoresis and chemiluminescence immunodetection imaging by a CCD camera using default settings: electrophoresis, 375 volts, 25 min; blocking, 5 min; primary antibody, 30 min; secondary antibody, 30 min; and camera exposure times, 1 sec to 120 sec. Compass software (ProteinSimple) was used to acquire and analyze the data and to generate gel images and chemiluminescence signal intensity values. Protein expression and phosphorylation levels are expressed as the chemiluminescence intensity area under the peak.

### 2.3. Mechanical Responses

Muscle strips were pretreated with 1 *μ*M carbachol for 5 min at 37°C in oxygenated KRB, and three 1 min washes with KRB, to remove any residual curariform neuromuscular paralytics [29]. Contractions were measured in static myobaths, with each muscle strip attached to a Fort 10 isometric strain gauge (WPI, Sarasota, FL, USA) [15]. Each strip was stretched to an initial resting force of ~0.6 g and then equilibrated for 30 min–45 min in 37°C oxygenated KRB. To measure the spontaneous phasic contractile activity, and the contractile responses to cholinergic neurotransmission, muscle strips were incubated with 100 *μ*M L-NAME and 1 *μ*M MRS2500 (to inhibit nitrergic and purinergic inhibitory motor neurons) prior to the delivery of square-wave pulses of electrical field stimulation (EFS) (0.3 ms duration), 150 V and 30 sec duration (supramaximal voltage; Grass S48 stimulator) [16]. Contractile activity was acquired and analyzed with AcqKnowledge 3.2.7 software (BIOPAC Systems, Santa Barbara, CA, USA).

### 2.4. Materials

Wes reagents were purchased from ProteinSimple. The primary antibodies used were rabbit anti-*γ*-actin (ACTG2) (GeneTex, GTX101794, 100-fold dilution); rabbit anti-myosin light chain kinase (MYLK) (Origene, TA347970, 400-fold dilution); rabbit anti-PP1*δ* (PPP1CB) (EMD Millipore, 07-1217, 100-fold dilution), Santa Cruz Biotechnology, Santa Cruz, CA, USA; rabbit anti-ROCK2 (sc-5561, 100-fold dilution); rabbit anti-CPI-17 (PPP1R14A) (sc-48406, 100-fold dilution); rabbit anti-pT38-CPI-17 (sc-17560-R, 50-fold dilution); rabbit anti-myosin light chain (MYL9) (sc-15370, 500-fold dilution); anti-pS19-MYL9 (sc-19849-R, 500-fold dilution); rabbit anti-MYPT1 (PPP1R12A) (sc-25618, 100-fold dilution); rabbit anti-pT853-MYPT1 (sc-17432-R; 100-fold dilution); and rabbit anti pT696/MYPT1 (sc-.017556; 200-fold dilution). Laboratory reagents were of analytical grade or better and purchased from Thermo Fisher, Sigma-Aldrich, and EMD Millipore.

### 2.5. Data and Statistical Analysis

Data are reported as means ± SD, median, and range. The relative expression and phosphorylation data is displayed as scatter plots of the mean ± SD of 9 smooth muscles. Data were analyzed using unpaired Student's *t*-test for comparisons between two groups and by nonparametric one-way analysis of variance (ANOVA) followed by Tukey's test for multiple-group comparisons. Differences were considered significant when *P* < 0.05. ^∗^*P* < 0.05, ^∗∗^*P* < 0.01, ^∗∗∗^*P* < 0.001, and ^∗∗∗∗^*P* < 0.0001. The “*n*” values refer to the number of samples used for each independent analysis. Graphs and statistical analyses were done using Prism 3.02 software (Jandel Scientific Software, San Jose, CA, USA). The peak area intensity values of pT853, pT38, and pS19 were divided by the MYPT1, CPI-17, and MYL9 peak area intensity values, respectively, from the same sample to obtain the ratio of phosphorylated protein to total protein. The peak area intensity values of MYLK, PP1*δ*, ROCK2, CPI-17, MYPT1, and MYL9 were divided by the peak area intensity values of *γ*-actin from the same sample to calculate protein expression relative to *γ*-actin expression. The digital lane views (bitmaps) of the immunodetected protein bands were generated by Compass software, with each lane corresponding to an individual capillary tube. The protein immunodetection figures were created from the digitized data using Corel Photo-Paint and CorelDRAW X4 (Corel Corp., Ottawa, Ontario, Canada). The area under the curve (AUC) of each contraction evoked by EFS was acquired using AcqKnowledge software.

## 3. Results

### 3.1. Ca^2+^ Sensitization and Contractile Protein Expression and Phosphorylation in Nondiabetic and Diabetic Gastric Fundus and Antrum Muscles

#### 3.1.1. *γ*-Actin Expression

**Figure 2:**
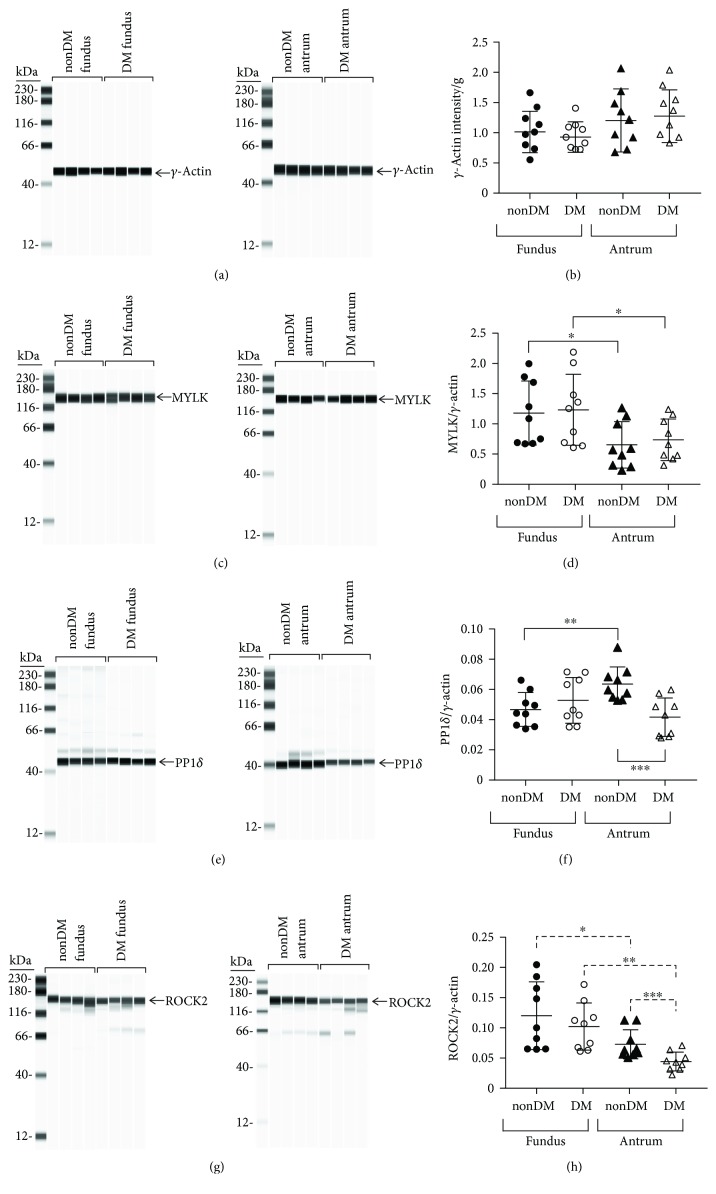
Protein expression levels in nondiabetic and diabetic gastric fundus and antrum smooth muscles. Representative immunoblots of (a) enteric *γ*-actin, 250 ng muscle lysate/lane, (c) MYLK, 1 *μ*g muscle lysate/lane, (e) PP1*δ*, 2.5 *μ*g muscle lysate/lane, and (g) ROCK2, 2.5 *μ*g muscle lysate/lane, expression in nondiabetic and diabetic fundus (left panel, *n* = 4, each group) and nondiabetic and diabetic antrum muscles (right panel, *n* = 4, each group). (b) Enteric *γ*-actin expression per g of nondiabetic and diabetic fundus and antrum muscles. (d) Smooth muscle MYLK expression relative to enteric *γ*-actin expression in nondiabetic and diabetic fundus and antrum smooth muscles. (f) PP1*δ* expression relative to enteric *γ*-actin expression in nondiabetic and diabetic fundus and antrum smooth muscles. (h) ROCK2 expression relative to enteric *γ*-actin expression in nondiabetic and diabetic fundus and antrum smooth muscles. ^∗^*P* < 0.05, ^∗∗^*P* < 0.01, and ^∗∗∗^*P* < 0.0001.

The expression levels of enteric smooth muscle *γ*-actin in nondiabetic (nonDM) and diabetic (DM) fundus and antrum muscles are similar (Figures 2(a) and 2(b)). The average values ± SD, median, and range of the *γ*-actin AUC intensities/g of nondiabetic and diabetic fundus muscle lysates were 1.02 ± 0.28, 0.99, 1.66–0.65 and 0.91 ± 0.21, 0.83, 1.41–0.81, respectively (Figure 2(b)). The average values ± SD, median, and range of the *γ*-actin AUC intensities/g of nondiabetic and diabetic antrum muscle lysates were 1.11 ± 0.51, 0.97, 2.04–0.87 and 1.12 ± 0.45, 0.92, 2.07–0.76, respectively (Figure 2(b)).

#### 3.1.2. MYLK Expression

MYLK expressions in nondiabetic and diabetic fundus muscles are similar (Figures 2(c) and 2(d)). The average ± SD MYLK/*γ*-actin ratios in nondiabetic and diabetic fundus muscles were 1.18 ± 0.53 and 1.23 ± 0.58, respectively (Figure 2(d)). The medians and ranges of the MYLK/*γ*-actin ratios were 1.08, 1.99–0.67 and 1.21, 2.18–0.69, and 0.66, respectively. MYLK expressions in nondiabetic and diabetic antrum muscles are also similar (Figures 2(c) and 2(d)). The average ± SD MYLK/*γ*-actin ratios in nondiabetic and diabetic antrum muscles were 0.65 ± 0.38 and 0.74 ± 0.34, respectively (Figure 2(d)). The medians and ranges of the MYLK/*γ*-actin ratios were 0.57, 1.23–0.31 and 0.66, 1.24–0.48, respectively. The average ± SD MYLK/*γ*-actin ratios in nondiabetic and diabetic fundus muscles are significantly higher than the corresponding average ± SD MYLK/*γ*-actin ratios in nondiabetic and diabetic antrum muscles, indicating that fundus muscles have a higher level of MYLK expression than antrum muscles have.

#### 3.1.3. PP1*δ* Expression

PP1*δ* expressions in nondiabetic and diabetic fundus muscles are similar (Figures 2(e) and 2(f)). The average ± SD PP1*δ*/*γ*-actin ratios in nondiabetic and diabetic fundus muscles were 0.05 ± 0.01 and 0.05 ± 0.015, respectively (Figure 2(f)). The medians and ranges of the PP1*δ*/*γ*-actin ratios were 0.05, 0.07–0.03 and 0.06, 0.07–0.03, respectively. In contrast, PP1*δ* expression in diabetic antrum muscles is significantly lower than in nondiabetic antrum muscles (Figures 2(e) and 2(f)). The average ± PP1*δ*/*γ* − actin ratios in diabetic and nondiabetic antrum muscles were 0.04 ± 0.01 and 0.06 ± 0.01, respectively (Figure 2(f)). The medians and ranges of the PP1*δ*/*γ*-actin ratios were 0.04, 0.06–0.03 and 0.06, 0.09–0.05, respectively. PP1*δ* expression in nondiabetic antrum muscles is higher than in nondiabetic fundus muscles (Figure 2(f)). However, PP1*δ* expressions in diabetic antrum and fundus muscles are similar, due to the lower level of PP1*δ* expression in diabetic antrum muscles.

#### 3.1.4. ROCK2 Expression

Although we found a wide range of ROCK2 expression levels in nondiabetic and diabetic fundus muscles, the average levels of ROCK2 expression were higher than the average expression levels in nondiabetic and diabetic antrum muscles (Figures 2(g) and 2(h)). The average ± SD ROCK2/*γ*-actin ratios in nondiabetic and diabetic fundus muscles were similar; 0.12 ± 0.05 and 0.11 ± 0.04, respectively (Figure 2(h)). The medians and ranges of the ROCK2/*γ*-actin ratios were 0.11, 0.21–0.06 and 0.11, 0.17–0.06, respectively. In contrast, diabetic antrum muscles have a significantly lower level of ROCK2 expression than nondiabetic antrum muscles have (Figures 2(g) and 2(h)). The average ± SD ROCK2/*γ*-actin ratios in nondiabetic and diabetic antrum muscles were 0.08 ± 0.02 and 0.04 ± 0.01, respectively. (Figure 2(h)). The medians and ranges of the ROCK2/*γ*-actin ratios were 0.06, 0.11–0.05 and 0.04, 0.07–0.02, respectively.

#### 3.1.5. MYPT1 Expression

**Figure 3:**
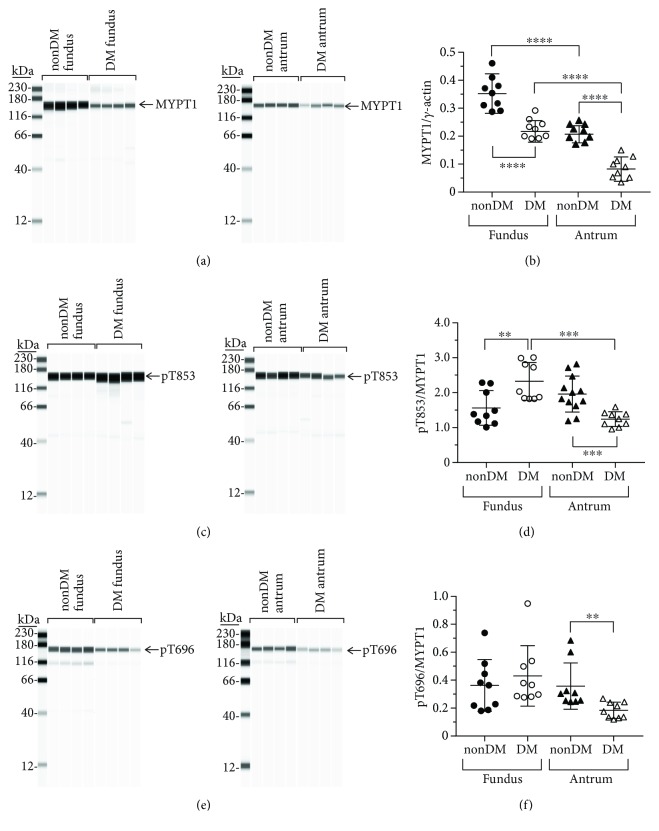
MYPT1 expression and T853 phosphorylation in nondiabetic and diabetic gastric fundus and gastric antrum smooth muscles. (a) Representative immunoblots of MYPT1 expression in nondiabetic and diabetic fundus (left panel, *n* = 4, each group) and nondiabetic and diabetic antrum muscles (right panel, *n* = 4, each group), 1 *μ*g muscle lysate per lane. (b) MYPT1 expression relative to enteric *γ*-actin expression in nondiabetic and diabetic fundus and antrum smooth muscles. (c) Representative immunoblots of phosphorylated T853 in nondiabetic and diabetic fundus (left panel, *n* = 4, each group) and nondiabetic and diabetic antrum muscles (right panel, *n* = 4, each group), 1 *μ*g muscle lysate per lane. (d) T853 phosphorylation relative to MYPT1 expression in nondiabetic and diabetic fundus and antrum smooth muscles. (e) Representative immunoblots of phosphorylated T696 in nondiabetic and diabetic fundus (left panel, *n* = 4, each group) and nondiabetic and diabetic antrum muscles (right panel, *n* = 4, each group), 1 *μ*g muscle lysate per lane. (f) T696 phosphorylation relative to MYPT1 expression in nondiabetic and diabetic fundus and antrum smooth muscles. ^∗∗^*P* < 0.01, ^∗∗∗^*P* < 0.001, and ^∗∗∗∗^*P* < 0.0001.

The expression levels of MYPT1, the inhibitory subunit of MLCP, in diabetic fundus and antrum muscles are significantly lower than the levels in nondiabetic fundus and antrum muscles (Figures 3(a) and 3(b)). The average ± SD MYPT1/*γ*-actin ratios in nondiabetic and diabetic fundus muscles were 0.35 ± 0.06 and 0.19 ± 0.03, respectively (Figure 3(b)). The medians and ranges of the MYPT1/*γ*-actin ratios were 0.31, 0.46–0.28 and 0.17, 0.26–0.16, respectively. For nondiabetic and diabetic antrum muscles, the average ± SD MYPT1/*γ*-actin ratios were 0.20 ± 0.03 and 0.08 ± 0.03, respectively (Figure 3(b)). The medians and ranges of the MYPT1/*γ*-actin ratios were 0.20, 0.24–0.17 and 0.07, 0.15–0.04, respectively.

#### 3.1.6. pT853/MYPT1 Ratios

The pT853/MYPT1 ratio in diabetic fundus muscles is significantly higher than the pT853/MYPT1 ratio in nondiabetic fundus muscles (Figures 3(c) and 3(d)). The average ± SD pT853/MYPT1 ratios in nondiabetic and diabetic fundus muscles were 1.56 ± 0.49 and 2.32 ± 0.54, respectively (Figure 3(d)). The medians and ranges of the pT853/MYPT1 ratios were 1.38, 2.86–1.01 and 2.03, 3.01–2.72, respectively. In contrast, the pT853/MYPT1 ratio in diabetic antrum muscles is significantly lower than the pT853/MYPT1 ratio in nondiabetic antrum muscles (Figures 3(c) and 3(d)). The average ± SD pT853/MYPT1 ratios in nondiabetic and diabetic antrum muscles were 2.16 ± 0.41 and 1.24 ± 0.21, respectively (Figure 3(d)). The medians and ranges of the pT853/MYPT1 ratios were 2.05, 2.81–1.72 and 1.25, 1.58–0.96, respectively.

#### 3.1.7. pT696/MYPT1 Ratios

The pT696/MYPT1 ratios in nondiabetic and fundus muscles are similar (Figures 3(e) and 3(f)). The average ± SD pT696/MYPT1 ratios in nondiabetic and diabetic fundus muscles were 0.36 ± 0.17 and 0.43 ± 0.21, respectively (Figure 3(f)). The medians and ranges of the pT696/MYPT1 ratios were 0.36, 0.74–10.18 and 0.37, 0.95–0.28, respectively. In contrast, the pT696/MYPT1 ratio in diabetic antrum muscles is significantly lower than the pT696/MYPT1 ratio in nondiabetic antrum muscles (Figures 3(e) and 3(f)). The average ± SD pT696/MYPT1 ratios in nondiabetic and diabetic antrum muscles were 0.36 ± 0.16 and 0.18 ± 0.05, respectively (Figure 3(f)). The medians and ranges of the pT696/MYPT1 ratios were 0.36, 0.68–0.24 and 0.18, 0.27–0.12, respectively.

#### 3.1.8. CPI-17 Expression

**Figure 4:**
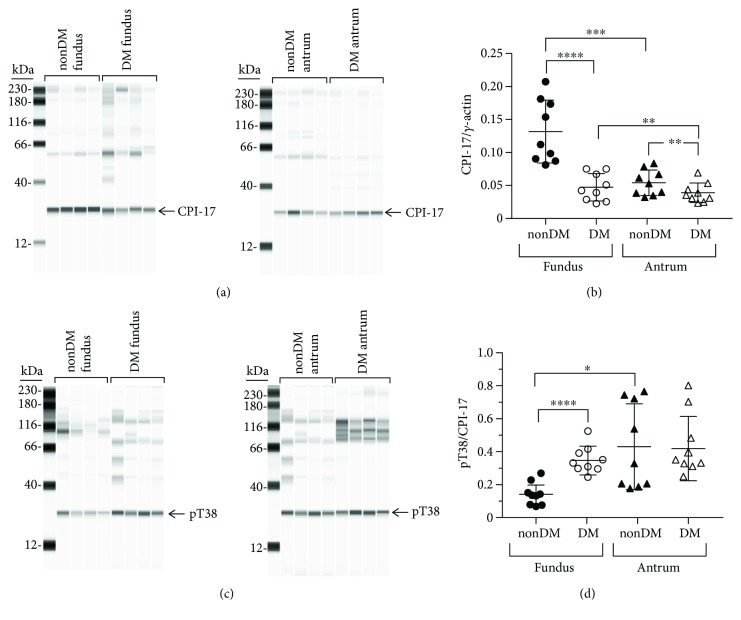
CPI-17 expression and T38 phosphorylation in nondiabetic and diabetic gastric fundus and antrum smooth muscles. (a) Representative immunoblots of CPI-17 expression in nondiabetic and diabetic fundus (left panel, *n* = 4, each group) and nondiabetic and diabetic antrum muscles (right panel, *n* = 4, each group), 1 *μ*g muscle lysate per lane. (b) CPI-17 expression relative to enteric *γ*-actin expression in nondiabetic and diabetic fundus and antrum smooth muscles. (c) Representative immunoblots of phosphorylated T38 in nondiabetic and diabetic fundus (left panel, *n* = 4, each group) and nondiabetic and diabetic antrum muscles (right panel, *n* = 4, each group), 1 *μ*g muscle lysate per lane. (d) T38 phosphorylation relative to CPI-17 expression in nondiabetic and diabetic gastric fundus and gastric antrum smooth muscles. ^∗^*P* < 0.05, ^∗∗^*P* < 0.01, ^∗∗∗^*P* < 0.001, and ^∗∗∗∗^*P* < 0.0001.

The expression levels of CPI-17, the inhibitor protein of MLCP, in diabetic fundus muscles are significantly lower than the expression levels in nondiabetic fundus muscles (Figures 4(a) and 4(b)). The average ± SD CPI-17/*γ*-actin ratios in nondiabetic and diabetic fundus muscles were 0.13 ± 0.05 and 0.05 ± 0.02, respectively (Figure 4(b)). The medians and ranges of the CPI-17/*γ*-actin ratios were 0.17, 0.21–0.08 and 0.04, 0.075–0.02, respectively. CPI-17 expression in diabetic antrum is slightly but still significantly lower than CPI-17 expression in nondiabetic antrum muscles (Figures 4(a) and 4(b)). The average ± SD CPI-17/*γ*-actin ratios in nondiabetic and diabetic antrum muscles were 0.05 ± 0.02 and 0.04 ± 0.01, respectively (Figure 4(d)). The medians and ranges of the CPI-17/*γ*-actin ratios in nondiabetic and diabetic antrum muscles were 0.06, 0.09–0.04, and 0.035, 0.07–0.02, respectively. CPI-17 expression in nondiabetic antrum muscles is higher than in nondiabetic fundus muscles (Figure 4(b)). However, CPI-17 expression in diabetic antrum and fundus muscles is similar, due to the lower level of PP1*δ* expression in diabetic antrum muscles.

#### 3.1.9. pT38/CPI-17 Ratios

The pT38/CPI-17 ratio in diabetic fundus muscles is significantly higher than the pT38/CPI-17 ratio in nondiabetic fundus muscles (Figures 4(c) and 4(d)). The average ± SD pT38/CPI-17 ratios in nondiabetic and diabetic fundus muscles were 0.14 ± 0.05 and 0.36 ± 0.08, respectively (Figure 4(d)). The medians and ranges of the pT38/CPI-17 ratios were 0.16, 0.27–0.13 and 0.33, 0.52–0.25, respectively. In contrast, although there was a wide range in the pT38/CPI-17 ratios from nondiabetic and diabetic antrum muscles, pT38/CPI-17 ratios in nondiabetic and diabetic antrum muscles are similar. The average ± SD pT38/CPI-17 ratios in nondiabetic and diabetic antrum muscles were 0.43 ± 0.26 and 0.42 ± 0.19, respectively (Figure 4(d)). The medians and ranges of the pT38/CPI-17 ratios were 0.33, 0.76–0.18 and 0.33, 0.81–0.25, respectively.

#### 3.1.10. MYL9 Expression

**Figure 5:**
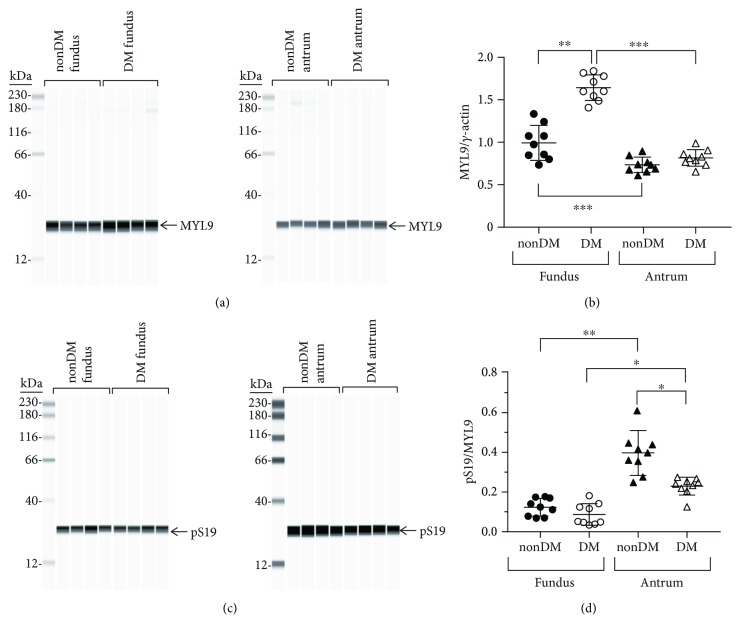
MYL9 expression and S19 phosphorylation in nondiabetic and diabetic gastric fundus and gastric antrum smooth muscles. (a) Representative immunoblots of MYL9 expression in nondiabetic and diabetic fundus (left panel, *n* = 4, each group) and nondiabetic and diabetic antrum muscles (right panel, *n* = 4, each group), 500 ng muscle lysate per lane. (b) MYL9 expression relative to enteric *γ*-actin expression in nondiabetic and diabetic fundus and antrum smooth muscles. (c) Representative immunoblots of phosphorylated S19 in nondiabetic and diabetic fundus (left panel, *n* = 4, each group) and nondiabetic and diabetic antrum muscles (right panel, *n* = 4, each group), 1 *μ*g muscle lysate per lane. (d) S19 phosphorylation relative to MYL9 expression in nondiabetic and diabetic fundus and antrum smooth muscles. ^∗^*P* < 0.05, ^∗∗^*P* < 0.01, and ^∗∗∗^*P* < 0.0001.

MYL9 expression in diabetic fundus muscles is significantly higher than MYL9 expression in nondiabetic fundus muscles (Figures 5(a) and 5(b)). The average ± SD MYL9/*γ*-actin ratios in nondiabetic fundus and antrum muscles were 0.99 ± 0.19 and 1.64 ± 0.14, respectively (Figure 5(b)). The medians and ranges of the MYL9/*γ*-actin ratios were 0.97, 1.33–0.73 and 1.61, 1.84–1.4, respectively. In contrast, MYL9 expression in diabetic antrum muscles is similar (Figures 5(a) and 5(b)). The average ± SD MYL9/*γ*-actin ratios in nondiabetic and diabetic antrum muscles were 0.73 ± 0.08 and 0.81 ± 0.09, respectively (Figure 5(b)). The medians and ranges of the MYL9/*γ*-actin ratios were 0.73, 0.89–0.61 and 0.81, 0.98–0.65, respectively.

#### 3.1.11. pS19/MYL9 Ratios

In contrast to the MYL9 expression levels, the pS19/MYL9 ratios in nondiabetic and diabetic fundus muscles are similar. The average ± SD pS19/MYL9 ratios in nondiabetic and diabetic fundus muscles were 0.12 ± 0.04 and 0.09 ± 0.05, respectively (Figure 5(d)). The medians and ranges of the pS19/MYL9 ratios were 0.12, 0.18–0.07 and 0.05, 0.18–0.03, respectively. Although there was a wide range in the pS19/MYL9 ratios from nondiabetic antrum muscles, the average ± SD pS19/MYL9 ratios from nondiabetic and diabetic antrum muscles are significantly higher than the pS19/MYL9 ratios in nondiabetic and diabetic fundus muscles (Figure 5(d)). In contrast to the similar pS19/MYL9 ratios in nondiabetic and diabetic fundus muscles, the pS19/MYL9 ratios in diabetic antrum muscles are significantly lower than the pS19/MYL9 ratios in nondiabetic antrum muscles (Figure 5(d)). The average ± SD pS19/MYL9 ratios in nondiabetic and diabetic antrum muscles were 0.40 ± 0.11 and 0.23 ± 0.04, respectively (Figure 5(d)). The medians and ranges of the pS19/MYL9 ratios were 0.40, 0.64–0.25 and 0.25, 0.27–0.13, respectively.

### 3.2. Spontaneous Contractile Activities of Nondiabetic and Diabetic Gastric Fundus and Antrum Muscles

**Figure 6:**
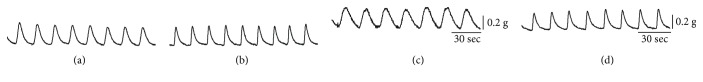
Spontaneous phasic contractile activities of nondiabetic and diabetic gastric fundus and antrum muscles. Representative traces of the spontaneous phasic contractions of muscle strips from (a) nondiabetic and (b) diabetic fundus muscles and (c) nondiabetic and (d) diabetic antrum muscles (*n* = 5 nondiabetic fundus and antrum muscles; *n* = 5 diabetic fundus and antrum muscles).

We found that the frequencies of the spontaneous phasic contractions from nondiabetic and diabetic gastric fundus and antrum muscles did not significantly differ from each other, regardless of age, sex, or diabetic status.  Figure 6 shows representative traces of the spontaneous phasic contractions from nondiabetic and diabetic gastric fundus and antrum muscles. The average ± SD cycles per min from nondiabetic and diabetic fundus muscles were 3.7 ± 0.6 and 3.3 ± 0.2, respectively (*n* = 5) (Figure 6(d)). Similarly, the average ± SD cycles per min from nondiabetic and diabetic antrum muscles were 3.8 ± 0.5 and 3.5 ± 0.2, respectively (*n* = 5).

### 3.3. Contractile Responses of Nondiabetic and Diabetic Gastric Fundus and Antrum Muscles to Cholinergic Motor Neuron Stimulation

**Figure 7:**
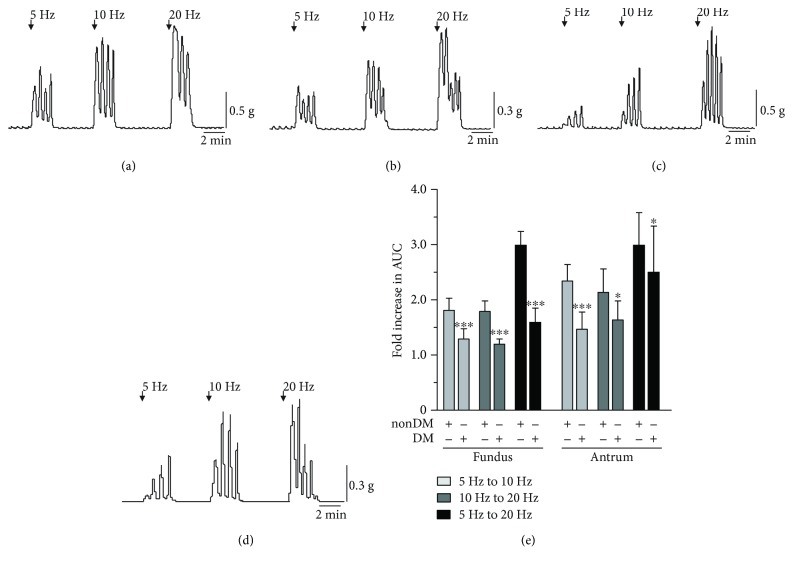
Contractile responses of nondiabetic and diabetic gastric fundus and antrum muscles to cholinergic motor neuron stimulation. Representative traces of the contractile responses of muscle strips to 5 Hz, 10 Hz, and 20 Hz EFS from (a) nondiabetic and (b) diabetic fundus muscles and (c) nondiabetic and (d) diabetic antrum muscles. (e) Mean fold increase in AUC ± SD of nondiabetic and diabetic fundus and antrum muscles stimulated with 5 Hz, 10 Hz, or 20 Hz EFS (*n* = 9). ^∗^*P* < 0.05 and ^∗∗∗^*P* < 0.0001.

Electrical field stimulation of gastric fundus and antrum muscles in the presence of L-NAME and MRS2500 was used to evoke contractions in response to cholinergic motor neurotransmission [16]. Because there was considerable variability in the absolute magnitudes of the contractile responses from different human stomach muscle samples, we measured contractile strength by comparing the fold increase in the area under the curve (AUC) of each contractile peak as the frequency of EFS was increased. The fold increases in AUC from diabetic fundus and antrum muscles were significantly lower compared to nondiabetic fundus and antrum muscles. As shown in Figure 7, for nondiabetic gastric fundus muscles, the AUC increased almost twofold as the EFS frequency increased from 5 Hz to 10 Hz and 10 Hz to 20 Hz (the average ± SD fold increase in AUC from 5 Hz to 10 Hz and 10 Hz to 20 Hz was 1.82 ± 0.21 and 1.80 ± 0.18, resp.). The average ± SD fold increase in AUC from 5 Hz to 20 Hz was 3.0 ± 0.24. For diabetic gastric fundus muscles, the average ± SD fold increase in AUC from 5 Hz to 10 Hz and 10 Hz to 20 Hz was 1.30 ± 0.18 and 1.21 ± 0.08, respectively. The average ± SD fold increase in AUC from 5 Hz to 20 Hz was 1.6 ± 0.25. Similarly, for nondiabetic gastric antrum muscles, the increase in AUC was around twofold as the EFS frequency increased from 5 Hz to 10 Hz and from 10 Hz to 20 Hz (the average ± SD fold increase in AUC from 5 Hz to 10 Hz and from 10 Hz to 20 Hz was 2.35 ± 0.29 and 2.14 ± 0.21, resp.). The average ± SD fold increase in AUC from 5 Hz to 20 Hz was 5.0 ± 0.88. For diabetic gastric antrum muscles, the average ± SD fold increase in AUC from 5 Hz to 10 Hz and from 10 Hz to 20 Hz was 1.48 ± 0.30 and 1.65 ± 0.33, respectively. The average ± SD fold increase in AUC from 5 Hz to 20 Hz was 2.51 ± 0.83.

## 4. Discussion

In this study, we compared the expression and phosphorylation of smooth muscle contractile proteins and proteins that regulate the Ca^2+^ sensitivity of the myofilaments, in gastric fundus and antrum muscles from obese nondiabetic and diabetic patients. We also compared the frequencies of spontaneous phasic contractions and the contractile responses to increasing frequencies of EFS of cholinergic motor neurons.

We found no differences in enteric smooth muscle *γ*-actin expression between nondiabetic and diabetic human fundus and antrum muscles, allowing for direct comparisons of protein expression relative to *γ*-actin expression. Relative to *γ*-actin, fundus muscles from nondiabetic obese patients have higher expression of MYLK, ROCK2, MYPT1, CPI-17, and MYL9, but lower PP1c*δ* expression, than antrum muscles. The pT853/MYPT1 and pT696/MYPT1 ratios in nondiabetic fundus and antrum muscles are similar, but the pT38/CPI-17 ratio is higher in antrum muscles. These findings suggest that nondiabetic antrum muscles may have lower levels of MLCP activity toward phosphorylated S19 of MYL9, which is consistent with supporting the strong phasic contractile activity of the gastric antrum that is required for the digestion of solid foods [31]. The higher pS19/MYL9 ratio in nondiabetic antrum muscles supports this conclusion.

Our findings that nondiabetic fundus muscles have higher expression of ROCK2, MYPT1, and CPI-17 than nondiabetic antrum muscles are similar to our findings in mouse gastric muscles [15]. However, in human fundus and antrum muscles, the pT853/MYPT1 ratios are similar, while it is lower in mouse antrum muscles, and the pT38/CPI-17 ratio in nondiabetic human antrum muscles is higher, while it is lower in mouse antrum muscles [15]. In addition, unlike human gastric muscles, we found that MYL9 expression is higher in mouse antrum muscles than in mouse fundus muscles [15]. Interestingly, the pS19/MYL9 ratio is higher in nondiabetic antrum muscles from both human and mouse [15]. Thus, both human and mouse antrum muscles have higher pS19/MYL9 ratios than fundus muscles, but for different reasons. In mouse antrum muscles, the higher pS19/MYL9 ratio likely is due to the higher MYL9 expression [15]. In human antrum muscles, MYL9 expression is lower, but the higher pS19/MYL9 ratio may result from lower MLCP activity due to the lower MYPT1 expression, and the higher pT38/CPI-17 ratio.

In diabetic fundus muscles, MYLK, ROCK2, and PP1*δ* expression were unchanged, MYPT1 and CPI-17 expression were decreased, and MYL9 expression was increased. The pT853/MYPT1 and pT38/CPI-17 ratios were increased, but the pT696/MYPT1 and pS19/MYL9 ratios were unchanged. The higher pT853/MYPT1 and pT38/CPI-17 ratios are likely due to the lower MYPT1 and CPI-17 expression levels, since ROCK2 and PP1*δ* expression is unchanged. The findings that the pT696/MYPT1 ratio is unchanged is consistent with recent observations demonstrating that MYPT1 T696 phosphorylation is constitutive and may be important for regulating the basal level of MLCP activity and only have a minor role in calcium-sensitized contraction [13]. Although the pT853/MYPT1 and pT38/CPI-17 ratios were increased, the decreased MYPT1 and CPI-17 expression suggests that the overall levels of phosphorylated MYPT1 and CPI-17 may be lower in diabetic fundus muscles. In contrast, the pS19/MYL9 ratio was unchanged, but MYL9 expression was increased, suggesting that the overall level of phosphorylated MYL9 is higher in diabetic fundus muscles. Since MYLK expression is unchanged, the activity of MLCP relative to MYLK may also be lower, which may allow MYLK to maintain the same pS19/MYL9 ratio in diabetic fundus muscles as in nondiabetic fundus muscles even with the increased MYL9 expression. However, since the constitutive pT38/CPI-17 ratio was increased, in response to cholinergic stimulation, a higher level of pT38 may be achieved, resulting in more inhibition of MLCP, which could explain the reduced fold increases in the AUC of diabetic fundus muscles to increasing frequencies of EFS.

In diabetic antrum muscles, only MYLK and MYL9 expression was unchanged; ROCK2, PP1c*δ*, MYPT1, and CPI-17 expressions were all decreased. The pT853/MYPT1, pT696/MYPT1, and pS19/MYL9 ratios were decreased in diabetic antrum muscles, but the pT38/CPI-17 ratio was unchanged. The decreased pT853/MYPT1 and pT696/MYPT1 ratios are consistent with the reduced ROCK2 expression. The decreased expression of MYPT1 and PP1c*δ* suggests that the overall level of MLCP is lower in diabetic antrum muscles. In addition, the decreased pT853/MYPT1 and pT696/MYPT1 ratios suggest that the overall level of phosphorylated MYPT1 is also lower in diabetic antrum muscles. Although the pT38/CPI-17 ratio is unchanged, CPI-17 expression is decreased, suggesting that the overall level of phosphorylated CPI-17 is also lower in diabetic antrum muscles. Also, although the overall level of MLCP may be lower, its overall level of activity may still be relatively high, due to the decreased levels of phosphorylated MYPT1 and CPI-17. The decreased pS19/MYL9 ratio in diabetic antrum muscles seems to support this conclusion. In addition, the magnitude of the increase in pT38 in response to cholinergic motor neurotransmission would be expected to be lower, due to the lower level of constitutive CPI-17 phosphorylation. MLCP would then be inhibited to a lesser extent, reducing the increase in pS19, which could explain the reduced fold increases in the AUC of diabetic antrum muscles to increasing frequencies of EFS.

We previously compared Ca^2+^ sensitization and contractile protein expression in gastric antrum muscles from 7- and 12-week-old wild-type C57BL/6J mice and *ob/ob* mice, a model of obesity and type 2 diabetes [32]. Our findings that MYLK expression is unchanged but ROCK2 expression is decreased in diabetic human antrum muscle are similar to our findings with *ob/ob* mice [20]. Although MYPT1 expression is slightly increased in *ob/ob* antrum muscles, and its expression is decreased in diabetic human antrum muscles, in both human and *ob/ob* antrum muscles the pT853/MYPT1 and pT696/MYPT1 ratios were decreased. Thus, in both *ob/ob* mouse and diabetic human antrum muscles, ROCK2 expression and MYPT1 T853 and T696 phosphorylation are decreased. Although CPI-17 expression is decreased in diabetic human antrum muscles and is unchanged in *ob/ob* antrum muscles, in both diabetic human and *ob/ob* antrum muscles the pT38/CPI-17 ratios were unchanged. These findings suggest that ROCK2 activity is selectively disrupted in diabetic antrum muscles. In diabetic human antrum muscles, MYL9 expression was unchanged but the pS19/MYL9 ratio was decreased. In *ob/ob* antrum muscles, MYL9 expression was unchanged at 7 weeks, but became higher at 12 weeks [20]. Similarly, the pS19/MYL9 ratio in *ob/ob* antrum muscles was unchanged at 7 weeks, but was reduced at 12 weeks of age [20]. This reduced pS19/MYL9 ratio at 12 weeks of age is consistent with the increased MYL9 expression at 12 weeks, and the decreased pT853/MYPT1 ratio, which would be expected to result in less MLCP inhibition.

Although we found differences in the expression and phosphorylation of Ca^2+^ sensitization and contractile proteins between gastric muscles from nondiabetic and diabetic patients, it is unknown whether obesity alone affects the expression and phosphorylation of these proteins. Studies in a variety of smooth muscles demonstrate that distension or stretch changes the expression and activities of several proteins [33–36]. These findings suggest that if obesity is associated with a higher gastric capacity, the gastric smooth muscles of obese patients may be stretched to a greater extent than in nonobese patients. However, studies of gastric capacity and gastric emptying have, in general, found that, functionally and anatomically, the stomachs of obese patients are similar to the stomachs of nonobese patients. Intragastric latex balloon measurements of compliance have found no significant differences in gastric capacity between nonobese and obese patients [37]. Three-dimensional ultrasound, MR imaging, and SPECT imaging of gastric volume also show that stomach volumes, dimensions, and weights are similar [37]. In addition, neither fasting gastric volume nor postprandial gastric volume is larger in obese compared to nonobese patients [38]. These findings suggest that gastric muscles in obese patients are not stretched to a significantly greater extent than they are in nonobese patients, suggesting that the differences in protein expression and phosphorylation we found in gastric muscles from obese patients are not due to mechanical or structural changes to the stomach. However, abnormal metabolic and endocrine alterations associated with obesity are well known [39, 40], and whether these might contribute to the observed protein expression and phosphorylation patterns are unknown. Additional studies comparing gastric muscles from nondiabetic obese and nonobese patients are necessary to address this question.

Long-standing diabetes adversely effects gastric motility, increasing the risk of developing gastroparesis [41]. The pathophysiology of diabetic gastroparesis, although not completely understood, is associated with a number of consistent findings, such as abnormalities in extrinsic and enteric neurons, interstitial cells of Cajal, smooth muscle cells, and immune cells [42]. Gastric smooth muscle dysfunction can develop, as indicated by disruptions in smooth muscle protein expression and an altered smooth muscle phenotype [43, 44]. Antral smooth muscle degeneration and fibrosis, with increased connective tissue stroma and thickened basal lamina, along with abnormalities in smoothelin-A expression are common findings in gastric biopsy specimens from patients with diabetic gastroparesis [28, 45]. These studies, and our findings reported here, suggest that diabetes contributes to detrimental changes in gastric smooth muscle Ca^2+^ sensitization and contractile protein expression, phosphorylation, and function, which may adversely affect gastric motility and contribute to the development of diabetic gastroparesis. Additional studies of Ca^2+^ sensitization and contractile protein expression and phosphorylation during agonist-evoked mechanical responses are underway to further characterize gastric muscles from nondiabetic and diabetic stomachs.

## Figures and Tables

**Table 1:** Clinical characteristics of the nondiabetic and diabetic patients.

Characteristic	Subjects without diabetes	Subjects with diabetes
Median age (range) (y)	47 (23–69)	49 (34–62)
Sex	10 female, 4 male	11 female, 3 male
BMI (median) (range) kg/m^2^	(40.23) (34–52)	(41.09) (34–48)
A1C (median) (range)	(5.4) (5.2–6.1)	(7.4) (6.3–9.5)

## Data Availability

The data used to support the findings of this study are available from Dr. Brian A. Perrino (bperrino@med.unr.edu) upon request.
